# HbA1c-defined dysglycemia among rural Bangladeshi adolescents: a population-based cross-sectional study

**DOI:** 10.1186/s12902-026-02472-y

**Published:** 2026-08-17

**Authors:** Haruko Takeuchi, S. M. Tafsir Hasan, Mohona Zaman, Kazi Nazmus Saqeeb, Sultana Yeasmin, Samar Kumar Hore, Masamine Jimba, Alfazal Khan

**Affiliations:** 1 Department of Community and Global Health, Graduate School of Medicine, The University of Tokyo, 7-3-1 Hongo, Bunkyo-ku, Tokyo, 113-0033 Japan; 2 Nutrition Research Division, International Centre for Diarrhoeal Disease Research, Bangladesh (icddr,b), 68, Shaheed Tajuddin Ahmed Sarani, Mohakhali, Dhaka, 1212 Bangladesh; 3 Department of Endocrinology, Universal Medical College, 74G/75, New Airport Road, Mohakhali, Dhaka, 1215 Bangladesh; 4 Jamjam Diagnostic Center, 47 N, Mugda, Mugda Road, Dhaka, 1214 Bangladesh; 5 Department of Community Medicine, Gonoshasthaya Samaj Vittik Medical College, Savar, Dhaka 1344, Bangladesh; Centre for Multidisciplinary Research, Gono Bishwabidyalay, Dhaka, 1344 Bangladesh; 6 Health Systems and Population Studies Division, International Centre for Diarrhoeal Disease Research, Bangladesh (icddr,b), 68, Shaheed Tajuddin Ahmed Sarani, Mohakhali, Dhaka, 1212 Bangladesh

**Keywords:** Adolescents, HbA1c-defined dysglycemia, Low-to-normal BAZ, Population-based study, Random sampling, Rural Bangladesh

## Abstract

**Background:**

Whereas dysglycemia among adolescents without overweight/obesity was reported in an urban hospital in Bangladesh during 2001–2002, rural epidemiological studies using fasting plasma glucose (FPG) and oral glucose tolerance test (OGTT) identified only type 1 and type 2 diabetes, with increased BMI reported as a risk factor. Thus, we aimed to determine the prevalence of dysglycemia and its association with body mass index-for-age z-score (BAZ) among adolescents in rural Bangladesh during a rapid nutritional transition.

**Methods:**

A population-based cross-sectional study was conducted from December 2017 to March 2018 in the Matlab Health and Demographic Surveillance System of the International Centre for Diarrhoeal Disease Research, Bangladesh (icddr,b) using a random sampling technique to recruit adolescents aged 10–19 years. A total of 291 adolescents underwent anthropometry and measurement of FPG and glycated hemoglobin (HbA1c). OGTT was performed on those with elevated FPG, elevated HbA1c, and/or positive postprandial glycosuria. Dysglycemia was defined according to the American Diabetes Association criteria of diabetes and prediabetes. Comparisons between adolescents with and without dysglycemia were performed using the Student’s t-test and the Mann–Whitney U test. Crude and adjusted odds ratios (cORs, aORs) for dysglycemia were calculated.

**Results:**

Dysglycemia was detected in 43 of 291 adolescents(14.8%; 95% CI 10.7–18.8%), with 68% of affected males aged ≤ 14 years. The median BAZ of the study population was −0.8. Risk for dysglycemia was associated with a family history of diabetes (aOR: 3.00; 95% CI 1.23 – 7.04; *p*= 0.017). Although overweight/obesity was more prevalent among adolescents with dysglycemia than among those without (16% vs 7%; *p* = 0.064), most adolescents with dysglycemia did not have overweight/obesity (36/43, 84%). HbA1c detected 36 of the 43 cases (84%), while no cases were identified by OGTT alone under the stepwise screening protocol, suggesting that screening strategies focused on FPG or overweight/obesity may fail to identify adolescents with dysglycemia in this population.

**Conclusions:**

HbA1c-defined dysglycemia was present among rural Bangladeshi adolescents and was not confined to overweight/obesity. These findings suggest that screening strategies based primarily on overweight/obesity may overlook at-risk individuals with low-to-normal BAZ.

**Supplementary Information:**

The online version contains supplementary material available at https://doi.org/10.1186/s12902-026-02472-y.

## Background

Diabetes mellitus is characterized by chronic hyperglycemia resulting from impaired insulin secretion and/or insulin resistance, representing a major global public health challenge [1–3]. These distinct states are clinically evaluated through fasting plasma glucose (FPG) and postprandial glucose levels, the latter commonly assessed using Oral glucose tolerance test (OGTT). Concurrently, South Asian nations are experiencing a sharp escalation in the prevalence of dysglycemia [4]. Emerging evidence indicates that South Asian populations manifest type 2 diabetes at lower BMI thresholds compared to their white counterparts [5]. This has been partly attributed to susceptibility among Asians, including Indians. Consequently, the World Health Organization (WHO) has established lower BMI cut-off points for metabolic risk assessment in Asian populations [6].

Although diabetes risk has conventionally been associated with advancing age and elevated BMI, its prevalence among children and adolescents has risen globally [7, 8]. Early-onset dysglycemia is particularly concerning, as it is characterized by accelerated disease progression, premature complications, and elevated cardiovascular risk, even at the prediabetic stage [9, 10]. These observations underscore the critical imperative for early identification and targeted prevention strategies during adolescence.

Since the turn of the twenty-first century, Bangladesh has undergone rapid economic development and a nutritional transition [11], which has substantially altered dietary habits and lifestyles. These shifts have precipitated a rising trend in overweight and obesity among pediatric populations [12]. However, within the context of these ongoing nutritional and epidemiological shifts—which are predominantly documented in urban sectors—it remains elusive whether non-overweight metabolic phenotypes persist among contemporary adolescents, particularly in rural Bangladesh [12]. Furthermore, adverse early-life nutritional exposures may exacerbate later metabolic risks, aligning with the Developmental Origins of Health and Disease framework [13]. Notably, recent documentation of dysglycemia among Bangladeshi adolescents with low-to-normal BMI suggests a potential heterogeneity in metabolic phenotypes.

Intrigued by this phenotypic diversity, prior hospital-based studies in Bangladesh have identified atypical forms of diabetes, including malnutrition-related and pancreatic phenotypes [14, 15] (summarized in Supplementary file 1 [14–20]). Conversely, rural, suburban, and urban epidemiological investigations utilizing oral glucose tolerance tests (OGTT) and fasting plasma glucose (FPG) have predominantly reported conventional type 1 and type 2 diabetes, reaffirming a strong correlation with higher BMI and older age [17, 18]. Thus, a critical knowledge gap remains regarding whether dysglycemia in adolescents without overweight or obesity is prevalent within rural Bangladeshi communities.

Driven by these dual transitions, the overall burden of diabetes is projected to escalate among Bangladeshi youth. Nevertheless, epidemiological data evaluating diabetes and dysglycemia among adolescents remain sparse, especially in rural settings, and evidence regarding the specific burden of dysglycemia across different BMI strata is limited. Given that early detection is paramount for mitigating long-term adverse outcomes and formulating effective public health interventions, updated population-based evidence is urgently required. Specifically, it remains to be elucidated whether dysglycemia clusters predominantly among adolescents with overweight/obesity or whether it extensively affects those with lower BMI-for-age z-scores (BAZ) in rural Bangladesh.

## Methods

### Aim, study design, study site, and the study participants

This study aimed to estimate the prevalence of dysglycemia and to examine its association with anthropometric status, specifically BAZ, among a randomly selected adolescent cohort in rural Bangladesh. Additionally, we explored the socio-demographic and lifestyle factors associated with dysglycemia.

A population-based cross-sectional study was conducted in rural Bangladesh using a random sampling technique to recruit adolescents aged 10–19 years. The study design was developed in collaboration with local researchers, reflecting the contextual understanding of health transitions in the study setting. The study was conducted in the Matlab health and demographic surveillance system (HDSS) maintained by the International Centre for Diarrhoeal Disease Research, Bangladesh (icddr,b) since 1966 [21]. The site was selected because it provides a well-defined population registry and infrastructure for epidemiological research, enabling population-based random sampling and field data collection. It is located in Chandpur District, approximately 55 km southeast of Dhaka, Bangladesh’s capital. The HDSS covers 142 villages with a population of about 220,000. The surveillance area comprises both an icddr,b service area and a government service area. The icddr,b service includes maternal and child health services for women of reproductive age and children under five years old through the Matlab Hospital and four sub-centres. Demographic and health information is collected regularly from residents in both areas through household visits by community health research workers every three months to gather information regarding births, deaths, marital status, and migration. The resulting population registry served as the sampling frame for the present study.

Eligible participants in the study were adolescents aged 10–19 years. A list of all adolescents aged 10 to 19 years who live in icddr,b service area was collected from the HDSS registration system at the beginning of the study. Then, a required number of adolescents was selected from that list by simple random sampling. Adolescents with conditions that precluded obtaining informed assent or parental consent were excluded. Adolescents with physician-diagnosed diabetes receiving anti-diabetic medication were included for prevalence estimation and questionnaire-based assessment, but were excluded from blood and urine testing.

### Sample size calculation

Sample size was calculated based on findings from a Bangladeshi study reported by Sayeed B et al. in 1997 [17]. The required sample size was calculated using the following formula:

1 $$n = {{{Z^2}P\left( {1 - P} \right)} \over {{d^2}}}$$

where n is the required sample size, Z is the Z value for a 95% confidence level (1.96), P is the expected prevalence (assumed to be 0.05), and d is the desired precision (0.025). Since the assumed prevalence was < 10%, P/2 was used in the calculation [22]. This yielded a required sample size of 292 participants. Assuming a 20% refusal rate and absenteeism, a total of 365 adolescents were planned for recruitment.

### Field data collection in a nearby household (EH) by two field organizers

Data were collected between December 2017 and March 2018 by one male and one female trained field organizer (FO). Participation required household contact and scheduling of a separate examination visit beforehand. Namely, selected adolescents were approached through household visits by trained field staff and were requested to attend a nearby household on a specified day. Then, adolescents who agreed to participate visited the designated household after an overnight fast. Participants were instructed to fast for 8–14 hours before the FPG test. They were only instructed to fast overnight before the FPG. Actual fasting duration was not recorded. That household was selected based on accessibility (walking distance) and the adolescents’ acceptability. Also, it should have been well-lit, well-ventilated, and have adequate workable space. That household was called the examining household (EH). Participants were requested to attend the assigned EH on a scheduled morning with a previously signed consent form. Minors and adolescents were accompanied by their guardians to the EH. After they provided the written informed consent (or assent with parental consent for minors), data collection began.The participants underwent a physical examination, including acanthosis nigricans [23, 24], anthropometric measurements, administration of a questionnaire, blood pressure measurement, urine glucose testing, capillary fasting plasma glucose (FPG) testing, and HbA1c testing. After the examination, they were asked to have breakfast provided by the study. They were further requested to stay in EH for 2 hours after breakfast to provide urine samples for glucose.

FPG was measured using capillary blood with a glucometer (Accu-Chek; Roche Diagnostics, Tokyo, Japan) [25]. HbA1c was measured using capillary blood with the cobas b 101 system (Roche Diagnostics, Tokyo, Japan), which is NGSP-certified [26]. Elevated FPG and HbA1c were defined as FPG ≥ 5.6 mmol/L, and HbA1c ≥ 5.7%.

For anthropometric and clinical measurements, height was measured using a portable stadiometer (Seca 213) and weight using a digital scale (Tanita HD-661). Waist and hip circumferences were measured using a non-stretchable measuring tape. Body mass index (BMI) was calculated as weight (kg)/height squared (m^2^). BMI-for-age z-scores (BAZ) were derived using the WHO 2007 growth reference for ages 5–19 years [27]. Blood pressure was measured using an automated manometer (Omron HEM-907 (JPN 2), Omron Healthcare, Japan).

A semi-structured questionnaire was used to assess diabetes-related factors, including socioeconomic status, smoking, family history of diabetes, symptoms suggestive of dysglycemia, and history of pregnancy. A WHO-based questionnaire on adolescent risk behaviors was also administered, including items on physical activity, exercise, sedentary behavior, sleep disturbance, and dietary habits [28].

Because universal OGTT was not feasible, it was offered only to selected adolescents.

### Data collection by an endocrinologist at Matlab Hospital

On a separate day at Matlab Hospital by an endocrinologist, confirmation of acanthosis nigricans and OGTT were performed. Participants were not instructed to follow any specific diet or physical activity regimen before testing. OGTT had been offered to adolescents with elevated HbA1c and FPG by ADA criteria, positive 2-hour postprandial glycosuria, and/or pregnancy at the first data collection. It was performed after an overnight fast with a glucose load of 1.75 g/kg body weight (maximum 75 g), and plasma glucose was measured 2 hours after the load. Positive postprandial glycosuria had been included for the selection criteria of OGTT because it might help identify potential IGT individuals from adolescents who tested negative for FPG and HbA1c by ADA criteria, although the sensitivity is low. Pregnant adolescents underwent OGTT but were excluded from the analytic sample.

### Definition of diabetes and prediabetes

Prediabetes and diabetes were defined according to the American Diabetes Association criteria [29]. Prediabetes was defined as FPG 5.6–6.9 mmol/L, HbA1c 5.7–6.4%, and/or 2-hour plasma glucose 7.8–<11.1 mmol/L by OGTT. Diabetes was defined as FPG ≥ 7.0 mmol/L, HbA1c ≥ 6.5%, and/or 2-hour plasma glucose ≥ 11.1 mmol/L.

### Statistical analysis

For statistical analysis, data were entered into and analyzed using IBM SPSS Statistics, versions 26 and 29.1 (IBM Japan, Tokyo, Japan). Analyses were restricted to non-pregnant adolescents. Descriptive statistics were calculated, and prevalence estimates were reported with 95% confidence intervals (CIs). Prevalence estimates are presented as percentages; the overall prevalence is reported to one decimal place, whereas subgroup prevalences were rounded to whole percentages due to smaller denominators. Continuous variables were compared using Student’s t-test or the Mann–Whitney U test, as appropriate, and categorical variables were compared using the χ^2^ test or Fisher’s exact test. Statistical significance was set at *p* < 0.05. We evaluated the diagnostic performance of postprandial urine glucose testing for identifying adolescents with impaired glucose tolerance (IGT), using OGTT as the reference standard. We tested the sensitivity, specificity, positive predictive value, and negative predictive value of 2-hour postprandial glycosuria with OGTT as the gold standard.

Crude and adjusted ORs for dysglycemia associated with anthropometric indicators and other risk factors were estimated using logistic regression analysis. Adjusted ORs were obtained from multivariable logistic regression models. The covariates included in each model are described in Tables 5 and 6. Multicollinearity was assessed before finalizing the regression models.

### Ethical approval and informed consent

This study was conducted in accordance with the ethical principles of the Declaration of Helsinki (as revised in Fortaleza, Brazil, October 2013). The research protocol was approved by the Research Ethics Committee of The University of Tokyo and by the Ethics Review Committee of icddr,b (approval details are provided in the Ethical Statements section). Written informed consent was obtained from participants aged 18 years or older. For participants under 18 years of age, written informed assent was obtained from the participants, along with written informed consent from their parents or legal guardians.

## Results

**Fig. 1:**
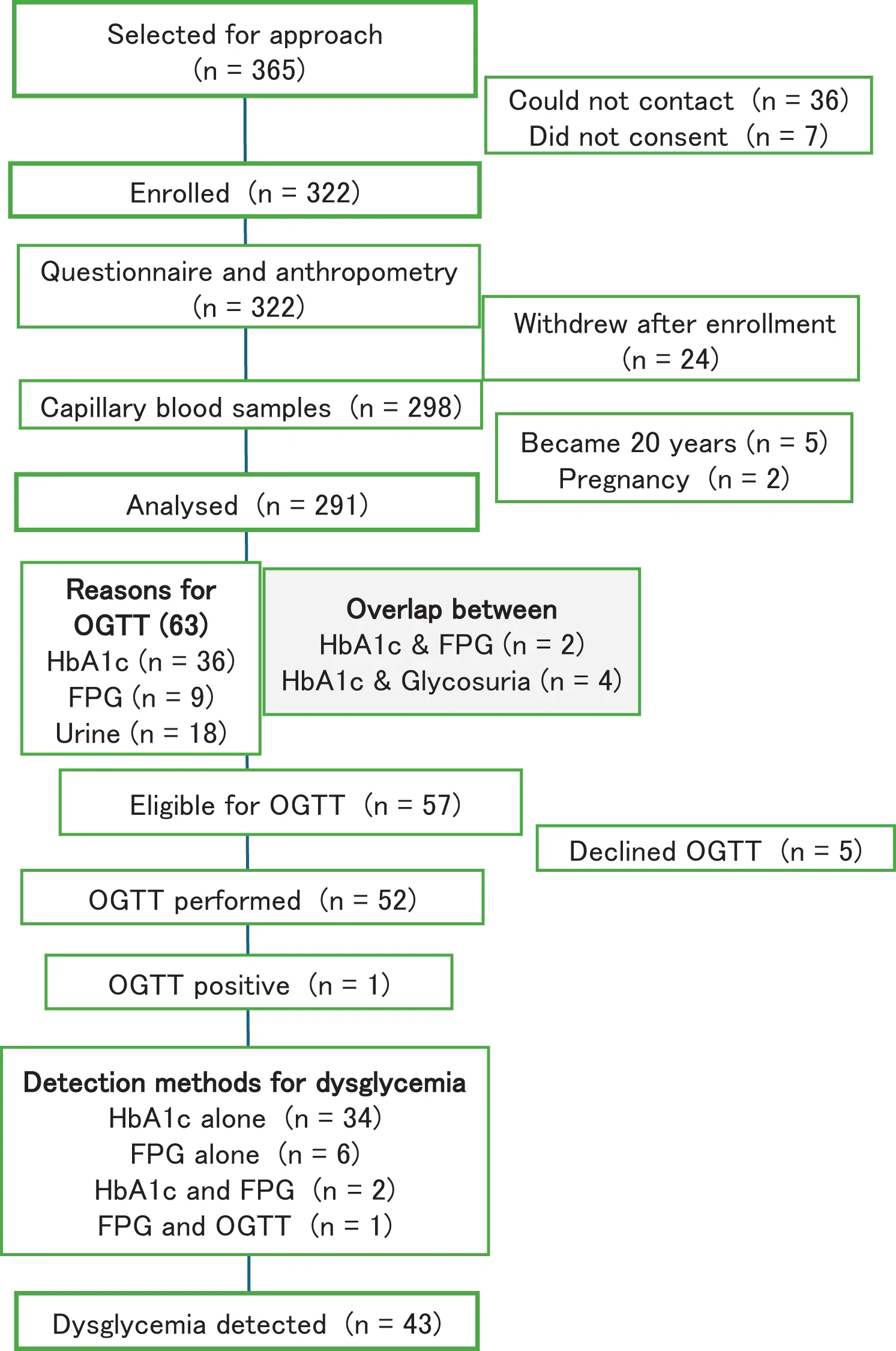
Flow diagram of participant selection and inclusion in the analysis. A total of 365 adolescents were approached; 322 were enrolled. Capillary blood samples were obtained from 298 participants, and 291 were included in the final analysis after exclusions

Figure 1 shows the flow diagram of the sampling procedure. A total of 365 adolescents were approached, of whom 322 were enrolled. Thirty-six could not be contacted, and seven were excluded because parental consent was not obtained. Questionnaire data and anthropometric measurements were collected from all 322 enrolled adolescents, and capillary blood samples were collected from 298, resulting in 24 withdrawals after enrollment. Among these 298 adolescents, seven were excluded: five had reached 20 years of age between sampling and data collection, and two were found to be pregnant. Thus, 291 adolescents were included in the final analysis.

Among the 291 adolescents, 36 had elevated HbA1c, 9 had elevated fasting plasma glucose (FPG), and 18 had 2-hour postprandial glycosuria, with 2 overlapping cases between HbA1c and FPG, and 4 overlapping cases between HbA1c and postprandial glycosuria. Of the 57 adolescents eligible for OGTT based on screening results, OGTT was performed in 52, as 5 declined; only 1 adolescent tested positive. A total of 43 adolescents were identified as having dysglycemia: 34 by HbA1c alone, 6 by FPG alone, 2 by HbA1c + FPG, and 1 by FPG + OGTT; none were identified by OGTT alone. The overall prevalence of dysglycemia was 14.8% (43/291; 95% CI 10.7–18.8%), and no diabetes was identified.

**Fig. 2:**
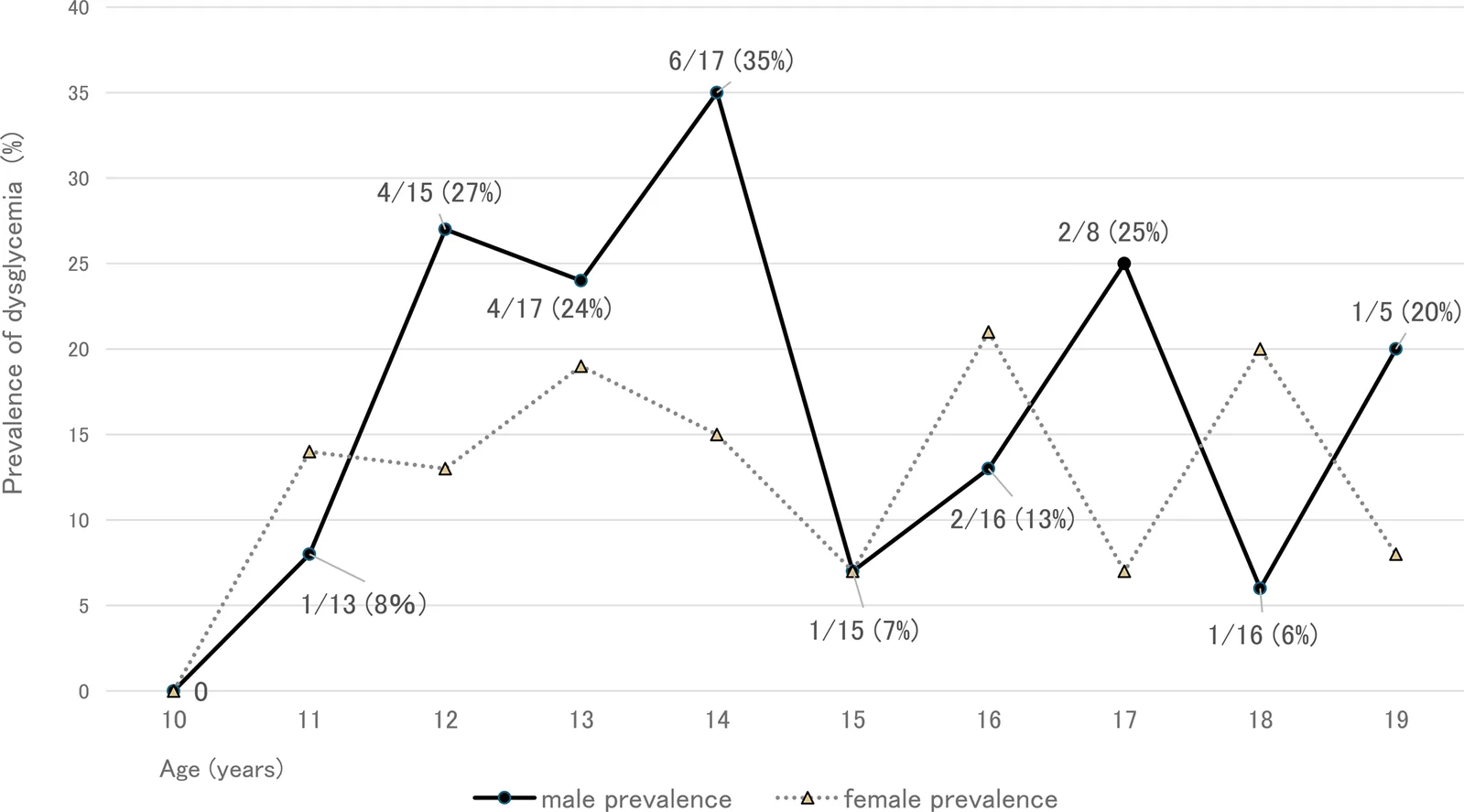
Age-specific prevalence of dysglycemia by sex among rural Bangladeshi adolescents. The vertical axis indicates the prevalence of dysglycemia (%) and the horizontal axis indicates age in years. Filled circles represent male adolescents and open triangles represent female adolescents. The age-specific numbers of male cases and total male participants (n/N) are shown in the figure; detailed counts for all age groups are provided in Table 1

Dysglycemia was found more among younger male adolescents; 15 of 22 male cases (68%) occurred at ages ≤ 14 years. In contrast, dysglycemia among females was more evenly distributed across ages. Age-specific prevalence was observed highest at the age of 14 years (35%, 6/17) (Table 1, Fig. 2). The study population was not generally overweight or obese (median BMI-for-age z-score (BAZ), −0.8) (Table 2). Median BAZ was −1.0 (−1.7 to −0.4) in males and −0.7 (−1.5 to 0.4) in females (Table 1).

**Table 1:** Age- and sex-specific prevalence of dysglycemia and BMI-for-age z-scores (BAZ) among adolescents with and without dysglycemia

Age	Male				Female			
	N	dysglycemia n (%)	BAZ (dysglycemia) median (IQR)	BAZ (normoglycemia) median (IQR)	N	dysglycemia n (%)	BAZ (dysglycemia) median (IQR)	BAZ (normoglycemia) median (IQR)
10	8	0 (0%)	-	−0.9 (−1.6 to −0.3)	12	0 (0%)	-	−1.0 (−1.5 to 0.2)
11	13	1 (8%)	0.4	−1.7 (−2.2 to −0.8)	22	3 (14%)	−1.1	−0.2 (−1.7 to 1.0)
12	15	4 (27%)	−0.4 (−2.1 to 1.3)	−1.1 (−1.8 to −0.5)	16	2 (13%)	1.6	−1.0 (−1.9 to −0.4)
13	17	4 (24%)	−1.5 (−1.6 to −0.7)	−0.7 (−1.2 to −0.1)	16	3 (19%)	−1.7	−1.0 (−2.0 to 0.44)
14	17	6 (35%)	−1.7 (−2.1 to −0.1)	−0.9 (−1.9 to −0.6)	20	3 (15%)	1.0	−0.7 (−1.9 to 0.4)
15	15	1 (7%)	−0.2	−0.7	14	1 (7%)	−1.1	−0.6 (−0.9 to 0.1)
16	16	2 (13%)	−1.4	−0.9 (−1.4 to −0.1)	19	4 (21%)	−1.3 (−2.6 to −1.1)	−0.5 (−1.4 to 0.6)
17	8	2 (25%)	−0.2	−2.3 (−3.1 to −1.7)	15	1 (7%)	2.8	−0.4 (−1.0 to 0.4)
18	16	1 (6%)	0.6	−1.0 (−1.7 to −0.5)	15	3 (20%)	−0.9	−0.0 (−1.6 to 0.4)
19	5	1 (20%)	−0.7	0.1 (−0.8 to 0.6)	12	1 (8%)	−0.7	−1.6 (−1.8 to −0.8)
Total	130	22 (17%)	−0.7 (−1.8 to 0.2)	−1.0 (−1.7 to −0.5)*	161	21 (13%)	−0.9 (−1.5 to 1.0)	−0.6 (−1.5 to 0.4)*
Total (Male)			−1.0 (−1.7 to −0.4)					
Total (Female)							−0.7 (−1.5 to 0.4)	

BAZ values are presented as median (interquartile range [IQR]). When the IQR could not be estimated due to small sample size, only the median is shown. IQR, interquartile range; *p = 0.029 (Mann–Whitney U test) for comparison of BAZ between males and females among adolescents with normoglycemia

**Table 2:** Characteristics of the study adolescents according to glycemic status and screening method

Variables	Total (n = 291)	dysglycemia (n = 43)	Normoglycemia (n = 248)	*p* **value**	Screened by FPG alone （n = 6）	Screened b HbA1c alone (n = 34)
Age (years) mean ± SD	14.3 ± 2.7	14.4 ± 2.3	14.3 ± 2.7	0.821	14.7 ± 2.4	14.3 ± 2.4
Male sex n (%)	130 (44.7%)	22 (51%)	108 (44%)	0.354		
BMI (kg/m^2^) mean ± SD	18.2 ± 3.3	18.8 ± 4.5	18.1 ± 3.1	0.322	19.6 ± 4.4	18.1 ± 3.6
BAZ median (IQR)	−0.8 (−1.6 to 0.1)	−0.8 (−1.7 to 0.6)	−0.8 (−1.6 to 0.0)	0.977	−0.6 (−1.7 to 1.5)	−0.9 (−1.7 to 0.2)
HAZ median (IQR)	−1.1 (−1.8 to −0.5)	−1.2 (−1.8 to −0.6)	−1.1 (−1.7 to −0.5)	0.474	−1.3 (−2.0 to −0.3)	−1.2 (−1.9 to −0.6)
Underweight (BAZ < −2) n (%)	35 (12.0%)	7 (16%)	28 (11%)	0.321		
Overweight/obesity (BAZ ≥ +1) n (%)	24 (8.2%)	7 (16%)	17 (7%)	0.064		
Stunting (HAZ < −2) n (%)	49 (16.8%)	9 (21%)	40 (16%)	0.507		
WHR mean ± SD	0.90 ± 0.05	0.88 ± 0.05	0.89 ± 0.05	0.506	0.90 ± 0.05	0.88 ± 0.05
Fasting glucose (mmol/L) mean ± SD	4.6 ± 0.5	4.9 ± 0.6	4.5 ± 0.5	0.001	5.7 ± 0.1	4.7 ± 0.5
HbA1c (%) mean ± SD	5.3 ± 0.3	5.7 ± 0.2	5.3 ± 0.2	<0.001	5.3 ± 0.1	5.8 ± 0.1

Data are presented as mean ± standard deviation (SD), median (interquartile range [IQR]), or number (%); p-values were calculated for comparisons between adolescents with and without prediabetes using Student’s t-test, Mann–Whitney U test, or χ² test, as appropriate.BAZ, body mass index-for-age z-score; FPG, fasting plasma glucose; HAZ, height-for-age z-score; HbA1c, glycated hemoglobin; OGTT, oral glucose tolerance test; WHR, waist-to-hip ratio.Values for FPG and HbA1c represent cases detected by each test alone; cases detected by both tests were excluded

Significant differences between adolescents with and without dysglycemia were observed for family history of diabetes (*p* = 0.008). Acanthosis nigricans (*p* = 0.059), overweight or obesity (*p* = 0.064), weight status (*p* = 0.068) (Table S2), television viewing time (*p* = 0.084) (Table S3), self-reported significant weight gain (*p* = 0.090) (Table S4), and years of education (*p* = 0.064) (Table S2) showed borderline significanc. No other associations were observed in assessed sociodemographic, behavioral, or clinical factors, including blood pressure and lifestyle-related variables. Comparison of these variables is shown in Tables S2, S3, and S4. The significant risk factors for dysglycemia by crude ORs were overweight or obesity (2.64; *p* = 0.044), family history (3.24; *p* = 0.008), and acanthosis nigricans (4.92 *p* = 0.042) (Table S5). However, when the variables were all included in a model, no variables remained significant (Table S5). In the reduced model excluding TV time (Table S6: Model A), acanthosis nigricans and years of education had borderline significance (aOR: 6.04 and 1.31; *p* = 0.073 and 0.076). When covariates were limited to only physical variables (Table S6: Model B), family history and acanthosis nigricans showed borderline significance (aOR 2.36 and 6.11; *p* = 0.071 and 0.075). When we further omitted the covariates with a *p*-value less than 0.100 (Table S6: Mode C), family history of DM became a significant risk factor (aOR 3.00; *p* = 0.017). Multicollinearity was assessed using Variance inflation factor (VIFs) in Model C: age 1.030, sex 1.081, overweight/obesity 1.303, family history 1.016, acanthosis nigricans 1.279, waist/hip ratio 1.125 (Table S6).

Most dysglycemic cases met the HbA1c criterion for dysglycemia (36/43; 84%, including overlapping cases), whereas no additional cases were identified by OGTT alone under the stepwise screening protocol. HbA1c was non-normally distributed in both groups; median HbA1c was 5.3% (IQR 5.1–5.5) in normoglycemic adolescents and 5.7% (IQR 5.6 to 5.8) in those with dysglycemia.

Postprandial glycosuria did not identify additional cases beyond those detected by other measures.

**Fig. 3:**
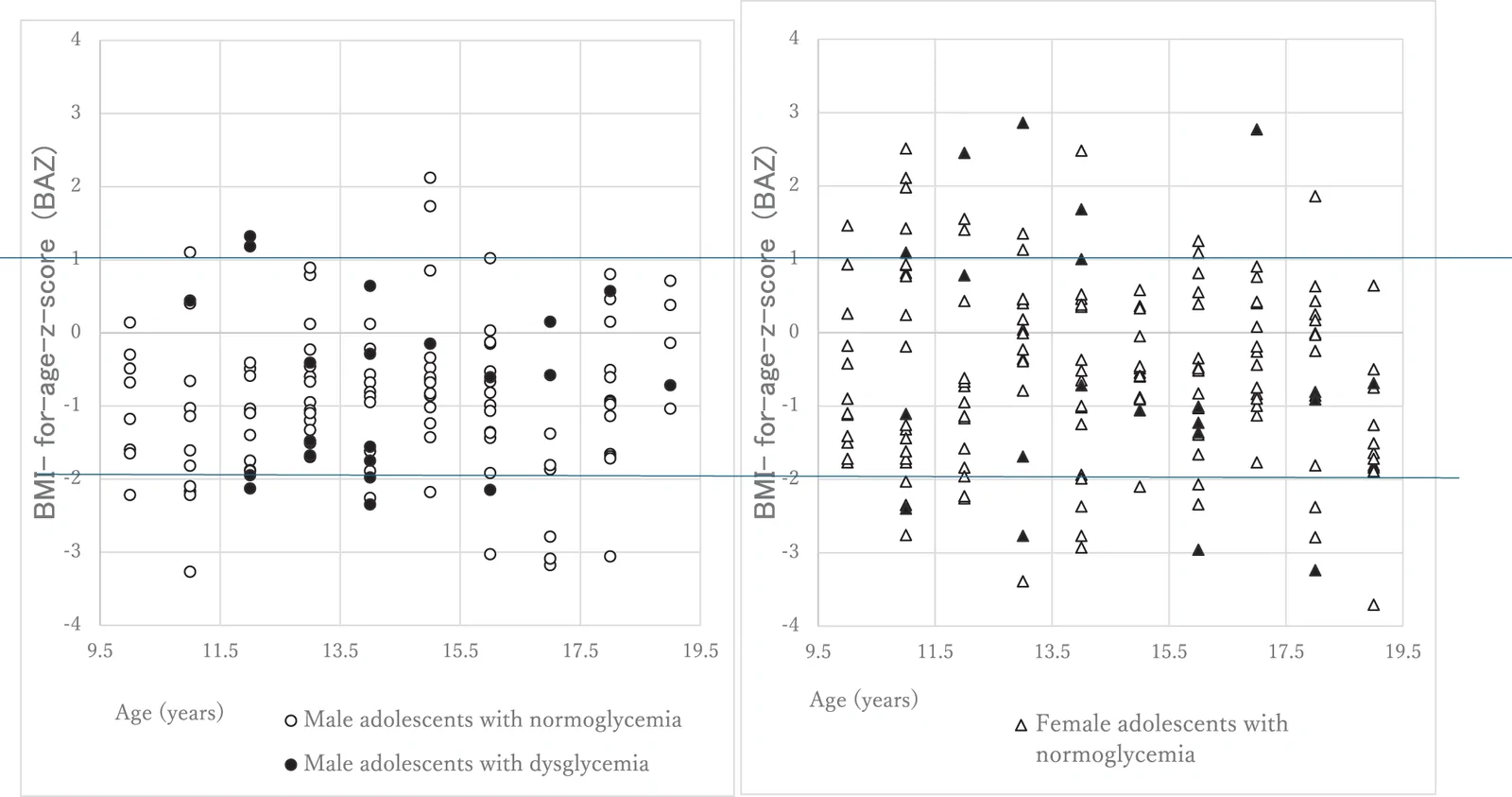
Age-specific distribution of BMI-for-age z-scores (BAZ) by sex andglycemic status among adolescents. The vertical axis indicates BAZ and the horizontal axis indicates age in years. Open circles and open triangles represent male and female adolescents with normoglycemia, respectively. Filled circles and filled triangles represent male and female adolescents with dysglycemia, respectively. The area between the horizontal lines at BAZ values of −2 and +1 represents the normal weight range

Among normoglycemic adolescents, BAZ was lower in males than in females (−1.0 vs −0.6; *p* = 0.011). Among those with dysglycemia, 3/22 males and 4/21 females were underweight (BAZ < −2), while 2/22 males and 5/21 females were overweight or obese (BAZ ≥ +1); most adolescents were within the normal BAZ range, and most dysglycemia was observed within this group (Fig. 3). Although the prevalence of overweight or obesity was higher among adolescents with dysglycemia than among those with normoglycemia (16% vs 7%; *p* = 0.064), most dysglycemia cases occurred among adolescents without overweight or obesity (Fig. 3).

## Discussion

In this population-based cross-sectional study with random sampling conducted in rural Bangladesh, HbA1c-defined dysglycemia was detected in 12.4% (36/291) of the adolescents. Elevated FPG was observed in 3.1% (9/291), whereas only one adolescent had a positive OGTT result. Most adolescents with HbA1c-defined dysglycemia had low-to-normal BAZ. These findings suggest that screening based solely on overweight or obesity may fail to identify a substantial proportion of adolescents with dysglycemia in this population.

Although the prevalence of overweight or obesity was higher among adolescents with dysglycemia than among those with normoglycemia, the majority (36/43, 84%) of adolescents with dysglycemia were not overweight or obese. These findings suggest that excess adiposity may contribute to dysglycemia in some adolescents, but it does not fully account for the observed distribution in this population.

OGTT was not performed in all participants, which may have led to an underestimation of the true prevalence, although most dysglycemia cases in the present study were identified by elevated HbA1c. This was because universal OGTT screening was not feasible in this population-based study conducted in a rural resource-limited setting. Instead, postprandial glycosuria was additionally used as a supplementary screening tool. Although urine glucose has limited sensitivity for identifying adolescents with positive OGTT results, the absence of additional OGTT-positive adolescents may suggest that overt postprandial dysglycemia was relatively uncommon in this population.

Additionally, the population-based study conducted in a rural, resource-limited setting led to the use of single point-of-care measurements without confirmatory testing, which may have resulted in some misclassification of glycemic status. Consequently, the estimated prevalence should be interpreted with caution. Further, the use of glucometer-based FPG diagnosis might have led to misclassification of dysglycemia.

Although HbA1c offers practical advantages in field settings, its interpretation requires caution because it can be influenced by non-glycemic factors such as iron deficiency, anemia, and hemoglobinopathies. Iron deficiency, anemia, and hemoglobinopathies have all been reported in Bangladesh and may have affected HbA1c levels in this population. However, iron deficiency and anemia are generally more prevalent among female adolescents, whereas dysglycemia in the present study clustered among younger male adolescents [30, 31]. In addition, hemoglobin E-related disorders are common in Bangladesh, but the age- and sex-specific distribution observed in this study may not be fully explained by hemoglobinopathies alone. Taken together, these findings suggest that the observed pattern of HbA1c-defined dysglycemia may not be entirely explained by anemia, iron deficiency, or hemoglobinopathies.

Adolescence is characterized by transient physiological insulin resistance associated with pubertal hormonal changes [32]. The clustering of dysglycemia among younger male adolescents may partly reflect this physiological insulin resistance. However, pubertal insulin resistance is generally compensated for by increased insulin secretion. Since additional metabolic stress, such as overweight or obesity, is often required for dysglycemia to develop, assessment of pubertal stage would have helped interpret this finding, but pubertal staging was not performed in the present study. Since in our target population overweight or obesity was uncommon and they reported moderate-to-vigorous physical activity. These findings may suggest an underlying metabolic vulnerability beyond overweight or obesity alone.

In the logistic regression analyses, overweight/obesity and acanthosis nigricans showed positive but non-significant associations with dysglycemia, whereas a family history of diabetes remained significantly associated with dysglycemia in Model C. However, given the small number of dysglycemia events (*n* = 43) and the wide 95% confidence intervals for the ORs, these findings should be considered exploratory and require confirmation in larger studies.The study area appeared to be undergoing nutritional transition during the study period, in which persistent undernutrition coexisted with the emerging presence of overweight and obesity potentially contributing to heterogeneous metabolic phenotypes. In such settings, improvements in food availability may not have been necessarily translated into adequate nutritional status or metabolic reserve at the population level. This transitional state may have contributed to heterogeneous metabolic phenotypes, in which dysglycemia arises not only from excess adiposity but also from developmental or nutritional vulnerability. This transitional state may have contributed to heterogeneous metabolic phenotypes, in which dysglycemia arises not only from excess adiposity but also from other developmental or nutritional factors that warrant further investigation.

These findings may suggest that dysglycemia in this population predominantly reflected mild chronic hyperglycemia rather than overt postprandial dysglycemia. The observed association with family history may be consistent with the possibility of underlying susceptibility related to glucose regulation.

The pattern of dysglycemia observed in the present study shares some characteristics previously reported in South Asian populations, including occurrence in adolescents without overweight or obesity and a possible contribution of familial susceptibility. However, the mechanisms underlying this phenotype remain unclear and warrant further investigation. 

### Strengths and limitations

 This study has several strengths, including a population-based design with random sampling, use of multiple glycemic measures, and provision of rare community-based data from rural Bangladesh.

Limitations include single point-of-care capillary glucose measurements without confirmatory repeat testing, the use of capillary blood rather than venous plasma, selective use of OGTT, lack of pubertal staging, the cross-sectional design, the potential influence of non-glycemic factors on HbA1c, the marginal elevation of HbA1c (median 5.7%), lack of direct measures of insulin secretion and insulin resistance, and limited generalizability beyond similar rural settings [31].

Since participants were sampled only from the icddr,b service area, findings may not be generalizable to other settings. However, including both surveillance areas might also have introduced additional heterogeneity because of systematic differences in health services between the icddr,b and government service areas.

These findings suggest that dysglycemia in this population may represent a heterogeneous metabolic phenotype. Further studies incorporating OGTT, measures of insulin secretion and insulin resistance, pubertal staging, and assessment of iron status and hemoglobin variants are warranted to clarify the mechanisms underlying dysglycemia in this population.

## Conclusion

HbA1c-defined dysglycemia was present among rural Bangladeshi adolescents and was not confined to overweight or obesity. These findings suggest that current screening strategies based primarily on anthropometric risk may overlook at-risk individuals in low-resource settings and underscore the need for context-specific approaches to early detection and prevention.

## Electronic supplementary material

Below is the link to the electronic supplementary material.

Supplementary Material 1

Supplementary Material 2

## Data Availability

The datasets used and/or analyzed during the current study are available from the corresponding authors on reasonable request.

## Abbreviations

BAZ: BMI-for-age z-score
BMI: Body mass index
CI: Confidence interval
DM: Diabetes mellitus
FCPD: fibrocalculous pancreatic diabetes
FPG: Fasting plasma glucose
HbA1c: Glycated hemoglobin
icddr,b: International centre for diarrhoeal disease research, bangladesh
IGT: Impaired glucose tolerance
IQR: Interquartile range
LMICs: Low- and middle-income countries
MMDM: Malnutrition-modulated diabetes mellitus
RAs: Research assistants
SD: Standard deviation
VIF: Variance inflation factor
WHO: World Health Organization
