# Supplementary material for: HbA1c-defined dysglycemia among rural Bangladeshi adolescents: a population-based cross-sectional study
Source: BMC Endocr Disord. 2026 Aug 17;26:370. doi: 10.1186/s12902-026-02472-y (PMC13757205; doi:10.1186/s12902-026-02472-y)
Supplement: Supplementary file 1 [file 12902_2026_2472_MOESM1_ESM.docx]

**Supplementary file 1A.** Epidemiological studies assessing ddysglycemia, and related outcomes among Bangladeshi adolescents.

| **Author**  **Published year** | **Country/Region** | **Setting** | **Population** | **Sample & sampling** | **Period / design** | **DM measurement / criteria** | **Outcomes** | **Key results (as reported)** | **How it differs from our study** | **Notes** |
| --- | --- | --- | --- | --- | --- | --- | --- | --- | --- | --- |
| Sayeed MA  1994 [16] | Bangladesh | Rural (Dohar thana, 5 villages) | ≥ 15y  1005 | .  Cluster sampling screening | NR / Cross-sectional | FPG  OGTT  WHO criteria  BMI | screening-related  NIDDM prevalence | sPHT 10.5%  dPHT 9%  NIDDM 2.1%  IGT 13.3%  BMI M/W  20.4/20.1 | **Part of adolescents**  **No HbA1c measurement**  BMI was a risk factor for INIDDM  HT・NIDDM・IGT increase with age | 1994 rural  BMI was  a risk factor for NIDDM |
| Sayeed MA  1997 [17] | Bangladesh | suburban | 6847  ≥ 15 y  271  < 20 y | Cluster-sampling | NR / Cross-sectional | FPG  OGTT  2₋ｈPG/WHO | Prevalence IGT  Prevalence of  NIDDM | IGT 7.5%  15-19 3.4%  DM 4.1%  15-19 2.6%  Risk factors  both M & F  BMI>22  Age  2-hPG  Weight | **(No) Rural**　（Suburban）  No data among  15－19 yr |  |
| Sayeed MA  2013 [18] | Bangladesh | Rural  Suburban Urban | 2151  adolescents  10-18 yrs | Cluster School sampling | NR / Cross-sectional | IFG  T2DM  AFG/WHO criteria  anthropometry | Prevalence of IFG, T2D, AFG | IFG 3.4%  T2DM 1.5%  AFG 4.9%  Rural prevalence  IFG 0.4%  T2D 0.4%  AFG 0.5% | **No population-based　study** | Females had higher dropout rate in secondarｙ school |
| SupplementaryTakeuchi H  (The present study) | Bangladesh | Rural | 291  10-19 yrs | Population-based  Random sampling | 2017-18  Cross-sectional | HbA1c  FPG  OGTT  BAZ  Postprandial glycosuria | Prevalence  Pre-DM | Prevalence: significantly higher in young males  Overweight OR for pre-DM was significant | **Population-based　study**  No risk factorse | Checked DOHaD |

**Supplementary file 1B. Hospital-based studies reporting phenotypes of dysglycemia in Bangladesh.**

| Mohsin F 2007 [14] | Bangladesh | Urban  Dhaka  DM Hospital | 88 new /125 DM adolescents  <1 8ys | whole | 2001-2002  Cross-sectional | Dr diagnosed DM | Detailed proportion  Among admitted DM patients | Total admission  125  T1D 38 (30%)  FCPD 37 (30%)  MMDM 48 (38%)  T2D 2 (1.6%) | Hospital based | MMDM and FCPD were 95% malnourished and poor |
| --- | --- | --- | --- | --- | --- | --- | --- | --- | --- | --- |
| Zabeen B  2017 [15] | Bangladesh | Dhaka  DM Hospital | 429 new  < 18yrs | Hospital DM cases | 2011-15 Comparative study  between  T1D &FCPD |  | FCPD | FCPD 106 (25%)  T1D 323 (75%) | FCPD was older  Had less FH  more cataracts  (9% vs 3%) | FCPD (25%) was not rare |
| Zabeen B  2019 [19] | Bangladesh | Dhaka DM Hospital | 100 consecutive new  < 22 yrs | Hospital DM cases | 2014.9- 2015.5 | By clinical diagnosis:  T1D 84/100  T2D 7/100  FCPD 9/100 | Age  F/M  Auto Ab  BMI z  DKA  C-peptide  DRA-DRB | T1D  2 peaks oolder  F>M  lower than Eurpop  T1D<T2D  Less DKA | **Population-based　study （No）**  **Rural (No)**  (Urban hospital) | **Atypical form of T1D**: **more common** than Europe |
|  |  |  |  |  |  |  |  |  |  |  |
| Zabeen B  2021 [20] | Bangladesh | Dhaka DM Hospital | 725 < 22 yr newly diagnosed cases | All cases | 2011-18  Retrospective repeat cross-sectional | Diagnosis of DM | DM annual  incidence  subtype | **T1D 66.5%**  T2D 28.3%  FCPD 1.9%  Others 3.3% | **Population-based　study （No）**  **Rural (No)**  (Urban hospital) | T1D 2/3  T2D incidence increase p=0.001 |

ADA: American diabetes	association	Auto Ab: auto antibody: BAZ: BMI for age z score	BMI: body mass index	DKA: diabetic ketoacidosis	DM diabetes mellitus	DOHaD: developmental origin of health and disease	DRA-DRB: HLA class II DRA/DRB genotype	F: female	FBG: fasting blood glucose	FCPD: fibrocalculous pancreatic diabetes	FH: family history	IGT: impaired glucose tolerance	M: male	MMDM: malnutrition-modulated diabetes mellitus	NR：not reported	T1D: type 1 diabetes mellitus	T2D: type 2 diabetes mellitus
