# Supplementary material for: HbA1c-defined dysglycemia among rural Bangladeshi adolescents: a population-based cross-sectional study
Source: BMC Endocr Disord. 2026 Aug 17;26:370. doi: 10.1186/s12902-026-02472-y (PMC13757205; doi:10.1186/s12902-026-02472-y)
Supplement: Supplementary file 2 [file 12902_2026_2472_MOESM2_ESM.docx]

**Table S2. Comparison of physical and socioeconomic characteristics between adolescents with and without dysglycemia**

BP: blood pressure

|  |  | dysglycemia | normoglycemia | *p* |
| --- | --- | --- | --- | --- |
|  |  | (n = 43) | (n = 248) |  |
| Age in years | Mean ± SD | 14.4±2.3 | 14.3±2.7 | 0.901 |
| Sex | Male | 22 (51%) | 108 (44%) | 0.354 |
| Systolic BP mmHg | Mean ± SD | 112±9 | 111 ±9 | 0.664 |
| Diastolic BP mmHg | Mean ± SD | 65 ±7 | 64 ± 6 | 0.461 |
| Acanthosis nigricans | yes | 3/39 (8%) | 4/240 (2%) | 0.059 |
| Years of education | Median (IQR) | 7 (5 to 8) | 6 (4 to 8) | 0.064 |
| Weight status Normal | yes | 29 (67%) | 203 (82%) | 0.068 |
| Underweight | yes | 7 (16%) | 28 (11%) |  |
| Overweight | yes | 4 (9%) | 13 (5%) |  |
| Obese | yes | 3 (7%) | 4 (2 %) |  |
| Overweight or obesity | yes | 7 (16%) | 17 (7%) | 0.064 |
| Family’s diabetes | yes | 27 (80%) | 124 (54%) | 0.008 |
| Household smoking | yes | 19 (44%) | 102 (41%) | 0.707 |
| Father’s years of education | Median (IQR) | 5 (2 to 9) | 5 (1 to 9) | 0.280 |
| Mother’s years of education | Median (IQR) | 5 (2 to 9) | 5 (3 to 8) | 0.783 |
| SES Deficit | yes | 11/42 (27%) | 55 (22%) | 0.513 |
| Balabce | yes | 25/42 (60%) | 137 (56%) |  |
| Surplus | yes | 6/42 (14%) | 54 (22%) |  |

SD: standard deviation	IQR: interquartile range

SES: socioeconomic status.

**Table S3. Comparison of adolescent risky behaviors for well-being between adolescents with and without dysglycemia**

|  | **Participation**  **prevalence** | Dysglycemia  n (%)  43 (100) | Normoglycemia  n (%)  248 (100) | *p =* | **Duration/**  **Amount**  unit | | Dysglycemia | | Normoglycemia | | *p =* | |  |
| --- | --- | --- | --- | --- | --- | --- | --- | --- | --- | --- | --- | --- | --- |
|  |  |  |  |  |  |  | Median (IQR) | | Median (IQR) | |  |  |  |
| Vigorous intensity work | | 5 (12%) | 31 (13%) | 1.000 | min/week | | 0 (0 to 0) | | 0 (0 to 0) | | 0.975 | |  |
| Moderate intensity work yes | | 17 (40%) | 121 (49%) | 0.262 | min/week | | 0 (0 to 100) | | 0 (0 to 120) | | 0.469 | |  |
| 10 minutes of transportation yes | | 37 (86%) | 192 (77%) | 0.232 | min/week | | 180 (80 to 240) | | 180 (27 to 300) | | 0.694 | |  |
| Exercise Vigorous sports yes | | 20 (47%) | 132 (53%) | 0.416 | min/week | | 0 (0 to 160) | | 30 (0 to 180) | | 0.670 | |  |
| Moderate intensity sports | | 27 (63%) | 159 (64%) | 0.868 | min/week | | 0 (0 to 0) | | 0 (0 to 0) | | 0.649 | |  |
| Sedentary hours total | | - | - | - | min/day | | 170 (120 to 240) | | 150 (90 to 240) | | 0.314 | |  |
| By TV | | - | - | - | min/day | | 60 (30 to 120) | | 60 (0 to 120) | | 0.084 | |  |
| By game | | - | - | - | min/day | | 0 (0 to 30) | | 10 (0 to 30) | | 0.752 | |  |
| By others | | - | - | - | min/day | | 60 (30 to 120) | | 60 (30 to 120) | | 0.862 | |  |
| Sleep at daytime | | - | - | - | min/day | | 0 (0 to 0) | | 0 (0 to 0) | | 0.205 | |  |
| Sleep at night | | - | - | - | min/day | | 480 (420 to 540) | | 480 (450 to 540) | | 0.707 | |  |
| Rice intake | | - | - | - | cup/day | | 42 (28 to 63) | | 42 (28 to 63) | | 0.997 | |  |
| Fruit intake yes | | 38 (88.4%) | 205 (82.7%) | 0.504 | servings/day | | 2 (1 to 4) | | 2 (1 to 4) | | 0.853 | |  |
| Vegetable intake yes | | 43 (100%) | 242 (97.6%) | 0.597 |  | servings/day | | 6 (3 to 88) | | 6 (3 to 9) | | 0.686 | |
| Junk food intake yes | | 40 (93.0%) | 231 (93.1%) | 1.000 | times/day | | 5 (3 to 7) | | 5 (3 to 7) | | 0.847 | |  |

SD: standard deviation	IQR: interquartile range

**Table S4. Comparison of self-reported symptoms commonly associated with DM between adolescents with and without dysglycemia**

|  | dysglycemia  n (%)  43 (100) | normoglycemia  n (%)  248 (100) | *P* = |
| --- | --- | --- | --- |
| Increased thirst | 13 (30.2%) | 70 (28%) | 0.788 |
| Increased appetite | 20 (47%) | 96 (38%) | 0.310 |
| Sweetened food favors no | 3 (7%) | 13 (5%) | 0.886 |
| some | 26 (61%) | 156 (63%) |  |
| Very much | 14 (33%) | 79 (32%) |  |
| Significant weight gain | 11 (29%) | 40 (17%) | 0.090 |
| Significant weight loss | 10 (25%) | 54 (23%) | 0.791 |
| Frequent urination at night | 10 (23%) | 66 (27%) | 0.644 |
| Blurring of vision | 10 (23%) | 36 (15%) | 0.147 |
| Loss of consciousness | 2 (5%) | 15 (6%) | 1.000 |

**Table S5. Crude odds ratios (ORs) and adjusted ORs for dysglycemia**

Adjusted ORs were obtained from multivariable logistic regression models including age, sex, waist/hip ratio, family history, overweight/obesity, acanthosis nigricans, self-reported significant weight gain, years of education, and TV watch time.

|  | cOR（95 % CI） | | *p* | aOR (95% CI) | | *p* |
| --- | --- | --- | --- | --- | --- | --- |
| Age in years per year | 1.01 | (0.90 – 1.15) | 0.820 | 0.92 | (0.69 – 1.23) | 0.574 |
| Sex Male/Female | 1.39 | (0.71 – 2.60) | 0.355 | 1.30 | (0.54– 3.15) | 0.556 |
| Overweight/obesity yes/no | 2.64 | (1.02–6.82) | 0.044 | 1.18 | (0.20– 6.33) | 0.845 |
| Family history yes/no | 3.24 | (1.35–7.73) | 0.008 | 2.02 | (0.78 – 5.24) | 0.147 |
| Waist/Hip per 1.0 | 0.10 | (0.00 – 90.27) | 0.504 | 0.11 | (0.00 – 1431.00) | 0.651 |
| Acanthosis nigricans yes/no | 4.92 | (1.06–22.88) | 0.042 | 5.30 | (0.65 – 37.50) | 0.106 |
| Significant weight gain yes/no | 1.95 | (0.89– 4.24) | 0.094 | 1.48 | (0.56 – 4.33) | 0.451 |
| Years of education per year | 1.13 | (0.99– 1.28) | 0.061 | 1.31 | (0.97 – 1.80) | 0.074 |
| Watch TV per min | 1.00 | (1.00– 1.01) | 0.219 | 1.00 | (1.00 – 1.01) | 0.444 |

**Table S6. Adjusted odds ratios (ORs) for dysglycemia**

**Model A, B, C**

| Model | | A | | | | | B | | | | C | | |
| --- | --- | --- | --- | --- | --- | --- | --- | --- | --- | --- | --- | --- | --- |
| Variables included | | aOR | | (95% CI) | *p* | | aOR | | (95% CI) | *p* | aOR | (95% CI) | *p* |
| Age in years | 0.93 | | (0.68 – 1.24) | | | 0.602 | | 1.13 | (0.96 – 1.32) | 0.129 | 1.09 | (0.94 – 1.26) | 0.247 |
| Sex Male/Female | 1.35 | | (0.51 – 3.23) | | | 0.506 | | 1.13 | (0.53 – 3.00) | 0.614 | 1.27 | (0.57 – 2.71) | 0.566 |
| Overweight/obesity | 1.29 | | (0.23 – 6.76) | | | 0.762 | | 1.53 | (0.30 – 7.88) | 0.607 | 1.59 | (0.33 – 7.45) | 0.560 |
| Family history | 2.05 | | (0.74 – 5.30) | | | 0.138 | | 2.36 | (0.93 – 6.01) | 0.071 | 3.00 | (1.23 – 7.04) | 0.017 |
| Acanthosis nigricans | 6.04 | | (0.81 – 43.20) | | | 0.073 | | 6.11 | (0.84 – 44.73) | 0.075 | 6.13 | (0.86 – 43.93) | 0.071 |
| Waist/Hip per 1.0 | 0.13 | | (0.00 – 1565.80) | | | 0.667 | | 0.04 | (0.00 – 540.14) | 0.515 | 0.04 | (0.86 – 326.30) | 0.488 |
| Significant weight gain | 1.54 | | (0.56 – 4.20) | | | 0.392 | | 1.53 | (0.57 – 4.41) | 0.399 |  |  |  |
| Years of education | 1.31 | | (0.97 – 1.78) | | | 0.076 | |  |  |  |  |  |  |

In the multivariate logistic regression analysis,

Model A included age, sex, waist/hip ratio, family history, overweight/obesity, acanthosis nigricans, self-reported significant weight gain, and years of education;

Model B included age, sex, waist/hip ratio, family history, overweight/obesity, acanthosis nigricans, and self-reported significant weight gain;

Model C included age, sex, waist/hip ratio family history, overweight/obesity, and acanthosis nigricans.

In model C. VIFs are age 1.030, sex 1.081, overweight/obesity 1.303, family history 1.016, acanthosis nigricans 1.279, waist/hip ratio 1.125.
